# µ-Theraphotoxin Pn3a inhibition of Ca_V_3.3 channels reveals a novel isoform-selective drug binding site

**DOI:** 10.7554/eLife.74040

**Published:** 2022-07-20

**Authors:** Jeffrey R McArthur, Jierong Wen, Andrew Hung, Rocio K Finol-Urdaneta, David J Adams

**Affiliations:** 1 Illawarra Health and Medical Research Institute, University of Wollongong Wollongong Australia; 2 School of Science, RMIT University Melbourne Australia; Columbia University United States; The University of Texas at Austin United States

**Keywords:** Ca_v_3.3, calcium channel, gating modifier toxin, Pn3a, T-type, Human

## Abstract

Low voltage-activated calcium currents are mediated by T-type calcium channels Ca_V_3.1, Ca_V_3.2, and Ca_V_3.3, which modulate a variety of physiological processes including sleep, cardiac pace-making, pain, and epilepsy. Ca_V_3 isoforms’ biophysical properties, overlapping expression, and lack of subtype-selective pharmacology hinder the determination of their specific physiological roles in health and disease. We have identified μ-theraphotoxin Pn3a as the first subtype-selective spider venom peptide inhibitor of Ca_V_3.3, with >100-fold lower potency against the other T-type isoforms. Pn3a modifies Ca_V_3.3 gating through a depolarizing shift in the voltage dependence of activation thus decreasing Ca_V_3.3-mediated currents in the normal range of activation potentials. Paddle chimeras of K_V_1.7 channels bearing voltage sensor sequences from all four Ca_V_3.3 domains revealed preferential binding of Pn3a to the S3-S4 region of domain II (Ca_V_3.3^DII^). This novel T-type channel pharmacological site was explored through computational docking simulations of Pn3a, site-directed mutagenesis, and full domain II swaps between Ca_V_3 channels highlighting it as a subtype-specific pharmacophore. This research expands our understanding of T-type calcium channel pharmacology and supports the suitability of Pn3a as a molecular tool in the study of the physiological roles of Ca_V_3.3 channels.

## Introduction

Voltage-gated calcium (Ca_V_) channels are activated by membrane depolarization and are involved in many physiological processes including contraction, secretion, neurotransmitter release, and gene expression (Catterall et al., 2005). In contrast to high voltage-activated (HVA) Ca^2+^ currents mediated by L-, N-, P/Q-, and R-type calcium channels that require large depolarizations, low voltage-activated (LVA) currents mediated by T-type Ca^2+^ channels are activated by small membrane depolarization and display distinctively faster activation and inactivation kinetics. T-type channel activation near resting membrane potentials generates low-threshold Ca^2+^ spikes responsible for the conspicuous burst firing and low-frequency oscillatory discharges observed in thalamic, olivary, and cerebellar neurons (Park et al., 2010; Dreyfus et al., 2010; Molineux et al., 2006; Lee et al., 2014). Thus, the biophysical properties of the T-type channels make them important regulators of cardiac and neuronal excitability (Perez-Reyes, 2003; Huguenard, 1996; Coulter et al., 1989) and therefore key pharmacological targets for the treatment of neurological and psychiatric disorders (Zamponi, 2016; Maksemous et al., 2022).

T-type calcium channels display small single-channel conductance (T stands for transient or tiny) (Perez-Reyes et al., 1998) and are encoded by the CACNA1G (Ca_V_3.1), CACNA1H (Ca_V_3.2), and CACNA1I (Ca_V_3.3) genes. The Ca_V_3s have similar channel activation and inactivation kinetics and are ubiquitously expressed in the nervous, neuroendocrine, reproductive, and cardiovascular systems (Perez-Reyes, 2003; Hansen, 2015). In heterologous expression systems, Ca_V_3.3-mediated currents display the slowest activation and inactivation kinetics and recover faster from inactivation (Kozlov et al., 1999), however, due to these relatively small differences, they are nearly indistinguishable from the other T-type isoform in native tissue. In the brain, abundant CACNA1I transcripts display remarkable regional distribution and appear to prevail in distal dendrites (Perez-Reyes, 2003; Lee et al., 1999), where Ca_V_3.3 currents mediate the major sleep-spindle pacemaker in the thalamus (Astori et al., 2011). Transgenic mice lacking Ca_V_3.3 channels display severe alterations to sleep-spindle generator rhythmogenic properties posing this T-type channel isoform as a critical target in the study of brain function and development (Astori et al., 2011). However, the expression of Ca_V_3.3 channels overlaps with that of Ca_V_3.1 and/or Ca_V_3.2, which together with the lack of robust, isoform-selective pharmacology has hampered the elucidation of their specific contributions to cellular physiology.

T-type calcium channels share the characteristic modular topology of other voltage-gated ion channels (VGICs) (Catterall et al., 2005) that consists of a voltage sensor (VS) module formed by transmembrane segments S1 through S4 and a pore module (PM) composed of the transmembrane segments S5 and S6 connected by a re-entrant pore loop. The VS controls channel opening in response to changes in membrane potential and the PM provides aqueous passage for ions across the lipid membrane. The tetrameric arrangement of four PMs lining the permeation pathway surrounded by four VSs in either swapped or non-swapped configuration enables VGIC function (for review, see Barros et al., 2019). In voltage-gated potassium (K_V_) and transient receptor potential (TRP) channels, each monomer (1 × VS + 1 × PM) is encoded by a core α-subunit; whereas in voltage-gated sodium (Na_V_) and Ca_V_ channels, the α-subunit contains the four homologous, but not identical, domains (DI-DIV) joined through large intracellular linkers (Catterall, 2000; Catterall et al., 2005).

Natural compounds that evolved to occlude ion channel’s PM or to interact with their VS are distinguished broadly as pore blockers and gating modifiers, respectively. In VGICs, the extracellularly exposed areas of the VS are pharmacological targets of neurotoxins and synthetic compounds where at least three distinct pharmacological sites have been described in Na_V_ channels (Catterall et al., 2007). Within the VS is a conserved S3b-S4 paddle motif which can be transplanted into the VS of other VGICs and retain toxin sensitivity (Bosmans et al., 2008). α-Scorpion toxins, sea-anemone toxins, and numerous spider toxins interact with Na_V_ channel site 3, located in the extracellular loop between DIV-S3 and DIV-S4 thereby interfering with the conformational changes that couple channel activation to fast inactivation (Hanck and Sheets, 2007). Binding of β-scorpion toxins to the S1-S2 and S3-S4 loops of domain II (site 4) shifts the voltage dependence of channel activation towards depolarized voltages reducing the maximal current at normal activation potentials. Lastly, pharmacological site 6 (located near site 3) is targeted by the δ-conotoxins that slow Na_V_ channel inactivation (Terlau et al., 1996). All these gating modifier peptides (GMPs) appear to ‘hold’ the VS in different conformations leading to their mechanism of modulation to be recognized as ‘VS trapping’ (Cestèle et al., 1998), a phenomenon also observed in the interaction of theraphotoxins with K_V_2.1 (Swartz and MacKinnon, 1997) and agatoxins with Ca_V_ channels (McDonough et al., 1997a).

Sequence and functional conservation between the VSs lead to promiscuous interactions between peptides and small molecules across VGIC families. Examples of these include ProTx-I (*Thrixopelma pruriens,* Na_V_/K_V_/Ca_V_/TRPA1) (Bladen et al., 2014; Bosmans et al., 2008; Gui et al., 2014; Middleton et al., 2002); ProTx-II (*T. pruriens,* Na_V_/Ca_V_) (Bladen et al., 2014; Middleton et al., 2002); Kurtoxin (*Parabuthus transvaalicus,* Na_V_/Ca_V_) (Chuang et al., 1998); Hanatoxin (*Grammostola spatulata*, K_V_/Na_V_/Ca_V_) (Bosmans et al., 2008; Li-Smerin and Swartz, 1998; Swartz and MacKinnon, 1997), and Pm1a (*Pelinobius muticus*, Na_V_/K_V_) (Finol-Urdaneta et al., 2022). Furthermore, small molecules such as capsaicin, capsazepine (TRPV1/K_V_/Ca_V_) (Caterina et al., 1997; Kuenzi and Dale, 1996; McArthur et al., 2019), and A803467 (Na_V_, Ca_V_) (Bladen and Zamponi, 2012) amongst others are known to interact across VGIC families.

Shared ancestry and sequence conservation within the voltage sensing machinery have been used to rationalize commonalities in structure, gating kinetics, and pharmacophores between Na_V_ and T-type channels (Bladen and Zamponi, 2012). Several Na_V_-active GMPs were shown to inhibit Ca_V_3.1 channels through interactions with the channel’s domain III (Ca_V_3.1^DIII^) (Salari et al., 2016); whereas the potent Na_V_1.7 inhibitor, µ-theraphotoxin Pn3a (*Pamphobeteus nigricolour*) (Deuis et al., 2017), also interacts with HVA Ca_V_ channels (McArthur et al., 2020).

In this study, we have used whole-cell patch clamp electrophysiology, mutagenesis, and computational docking to probe Pn3a’s interactions with the LVA Ca_V_3 channels. Our results support the use of Pn3a as a molecular tool for the study of Ca_V_3.3-mediated currents in native cells and highlight a previously unrecognized pharmacophore that may enable selective targeting of T-type channel isoforms.

## Results

### Pn3a selectively inhibits Ca_V_3.3 channels

**Figure 1.**
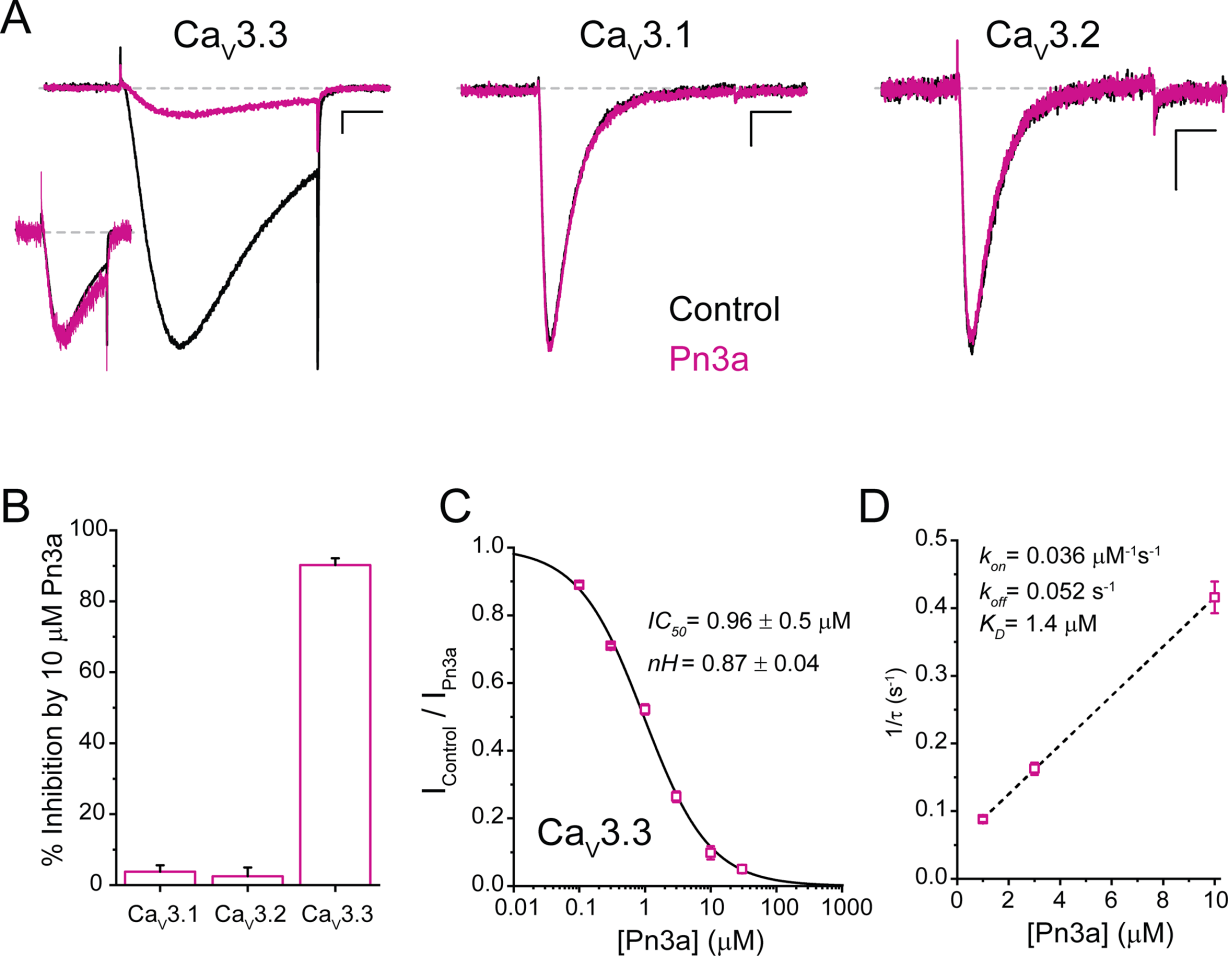
Pn3a preferentially inhibits human Ca_V_3.3-mediated Ca^2+^ currents. (**A**) Ca_V_3.3 (left), Ca_V_3.1 (middle), and Ca_V_3.2 (right) currents elicited by 100 ms step depolarization to −20 mV (Vh −90 mV, 0.2 Hz) in the absence (control, black) and presence of 3 µM Pn3a (pink). Scale bars: 0.2 nA, 20 ms. The inset shows scaled Ca_V_3.3 currents in control and in the presence of Pn3a with similar kinetics. (**B**) Bar graph summarizing % inhibition by 10 µM Pn3a of the three Ca_V_3 isoforms. (**C**) Concentration-response curve for Pn3a inhibition of Ca_V_3.3 currents. (**D**) Kinetics of Pn3a inhibition of Ca_V_3.3. K_obs_ was determined at three concentrations and fit with a linear equation where K_obs_ = k_on_ ∙ [Pn3a]+k_off_. Data shown as mean ± SEM (n=5). Figure 1—source data 1.Pn3a inhibition of T-type calcium channels.

Functional assessment of Pn3a activity was examined on depolarization-activated calcium currents (I_Ca_) through the human T-type calcium channel isoforms: Ca_V_3.1, Ca_V_3.2, and Ca_V_3.3. Whole-cell currents were elicited by a 100 ms test pulse to −20 mV from a holding potential (Vh) of −90 mV at a frequency of 0.2 Hz and recorded at room temperature (20–22°C, Figure 1A). Pn3a (10 µM) strongly inhibited Ca_V_3.3-mediated currents (90.2% ± 1.9%, n=5) with negligible effects over the two other T-type isoforms (Ca_V_3.1: 3.8% ± 1.8%, n=5; Ca_V_3.2: 2.5% ± 2.5%, n=5) (Figure 1A and B). These results indicate that Pn3a has >100-fold preference for Ca_V_3.3 over the other highly homologous Ca_V_3 isoforms. Scaled Ca_V_3.3 macroscopic currents recorded in the absence (control) and presence of Pn3a (3 µM) display similar macroscopic activation kinetics (τ_act, control_ = 5.95 ± 0.54 ms vs. τ_act, Pn3a_ = 6.37 ± 0.44 ms; p=0.32, n=5, paired t-test), whereas Pn3a slowed macroscopic inactivation kinetics (τ_inact control_ = 39.91 ± 8.25 ms vs. τ_inact, Pn3a_ = 60.25 ± 16.02 ms; p=0.04, n=5, paired t-test) (representative currents provided in the inset, Figure 1A). The preferential actions of Pn3a highlight its potential as a molecular probe to isolate and study the contribution of Ca_V_3.3 currents in native cells.

Pn3a inhibitory potency against Ca_V_3.3 currents was assessed at increasing peptide concentrations from which a concentration-response curve was built (Figure 1C). Fit to a standard Hill equation rendered an IC_50_ value of 0.96±0.05 µM (nH 0.87±0.04, n=5 per concentration) for the inhibition of Ca_V_3.3 channels. This Hill coefficient is consistent with a 1:1 stoichiometry between Pn3a toxin and Ca_V_3.3 channels.

The change in Ca_V_3.3 peak current amplitude during Pn3a *washin* and *washout* enables the assessment of Pn3a binding to Ca_V_3.3 channels. The inhibition of Ca_V_3.3 I_Ca_ by Pn3a was mono-exponential with progressively faster time constants (τ_obs_) at increasing peptide concentration (Figure 1D). The on- and off-rate constants (k_on_ and k_off_) were determined from the fit to the linear plot of 1/τ_obs_ vs. [Pn3a] (Figure 1D, n=5 per concentration), where k_on_ is the slope and k_off_ is the y-intercept. The linear regression line results in a k_on_ of 0.036 µM^–1^ s^–1^ and k_off_ of 0.052 s^–1^ which yield a K_D_ of 1.4 µM, in close agreement with the IC_50_ value obtained (Figure 1C). k_off_ was confirmed by fitting the *washout* to a single exponential (k_off_ = 0.055 ± 0.002 s^–1^). Hence, Pn3a inhibits Ca_V_3.3-mediated currents without apparent actions on Ca_V_3.1 or Ca_V_3.2 exposed to up to 10 µM peptide.

### Pn3a modifies the gating of Ca_V_3.3

**Figure 2.**
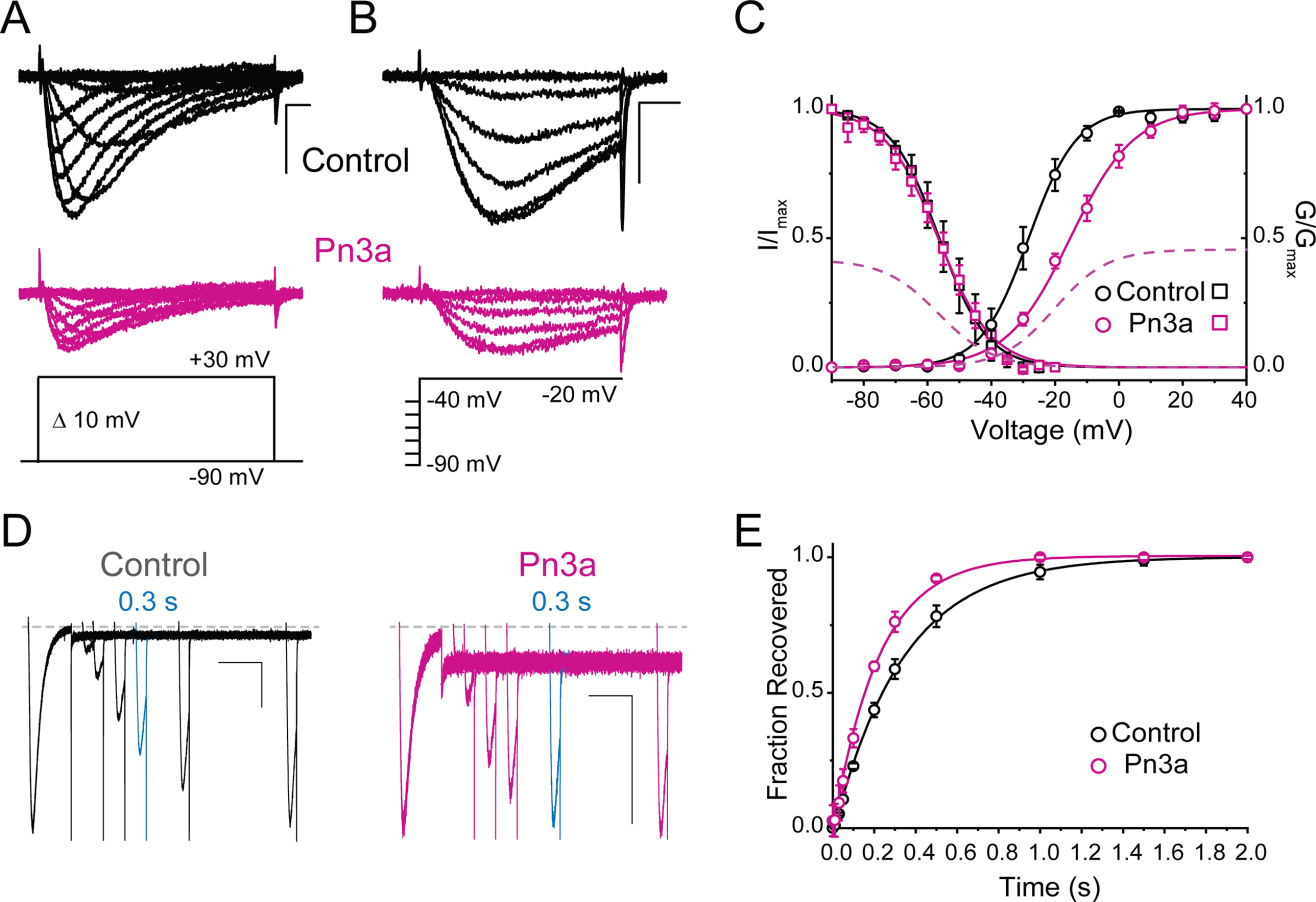
Pn3a produces a depolarizing shift in the voltage dependence of activation and speeds up recovery from inactivation of Ca_V_3.3. (**A–C**) Effect of Pn3a on the voltage dependence of Ca_V_3.3. Representative currents from (**A**) activation or (**B**) steady-state inactivation protocols, in the absence (top: control, black) and presence of 3 µM Pn3a (middle: Pn3a, pink) using the standard protocols (bottom). Scale bars: 0.5 nA, 10 ms. (**C**) Activation (circles) and steady-state inactivation (squares) relationships for Ca_V_3.3 in the absence (black) and presence of 3 µM Pn3a (pink). Dashed lines represent non-normalized activation and inactivation curves of Pn3a. (**D**) Representative recovery from inactivation currents in control (left) and presence of 3 µM Pn3a (right) (trace shown in blue highlights the current recovered after 0.3 s). Scale bars: 0.2 nA, 200 ms. (**E**) Recovery from inactivation in the absence (black) and presence of 3 µM Pn3a (pink). Data shown as mean ± SEM (n=5). Figure 2—source data 1.Pn3a voltage effects o activation, steady-state inactivation, and recovery from inactivation.

The voltage dependence of Ca_V_3.3 activation, deactivation, inactivation, and recovery from inactivation were investigated in the absence and presence of Pn3a (3 µM) (Figure 2 and Table 1). Ca_V_3.3 activation is shifted ~13 mV to more depolarized potentials (control V_0.5_ = −28.3±0.4 mV, n=5, vs. Pn3a V_0.5_ = −15.3±0.4 mV, n=5; p<0.0001), indicating that a stronger depolarization is required to enable channel opening in the presence of Pn3a (Figure 2A and C). The voltage dependence of Ca_V_3.3 steady-state inactivation (SSI) was not affected by exposure to the spider peptide (control V_0.5_ = −56.3±0.3 mV, n=5, vs. Pn3a V_0.5_ = −56.6±0.4 mV, n=5, p=0.57, Figure 2B–C). Consistent with the observed slowing of inactivation, Ca_V_3.3-mediated currents inactivated by a 200 ms pre-pulse to –20 mV recovered faster in the presence of Pn3a (τ=0.22 ± 0.01 s, n=5) than under control conditions (τ=0.34 ± 0.01 s, n=5; p<0.0001) (Figure 2D–E), suggesting a potential Pn3a-dependent destabilization of the inactivated state.

**Figure 2—figure supplement 1.**
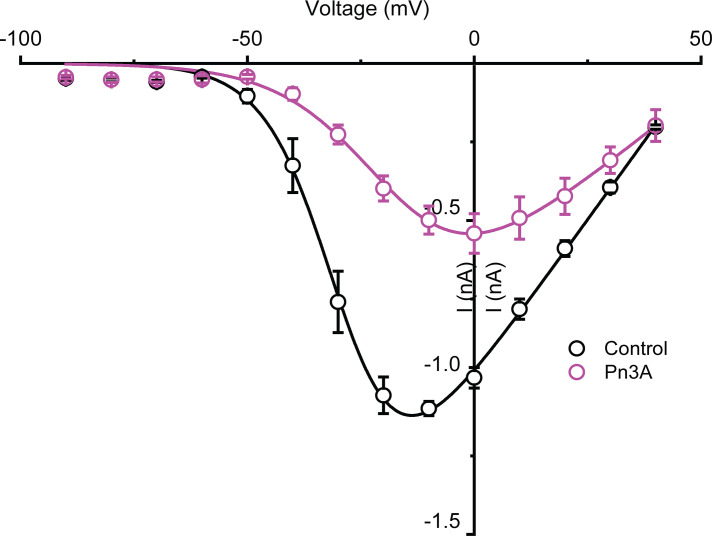
Current-voltage relation of control (black) and 3 µM Pn3a (pink) inhibition of human Ca_V_3.3.

**Table 1.** Activation and inactivation values of Ca_V_3.3 and K_v_1.7-Ca_V_3.3^DI-IV^ chimers in the absence (control) and presence of Pn3a (3 µM for Ca_V_3.3; 1 µM for K_V_1.7-Ca_V_3.3^D1-IV^ chimeras).

		Control Pn3a	
**hCa_V_3.3**	**V_0.5_ activation**	−28.3±0.4 mV (5)	−**15.3±0.4 mV (5)** *
	ka **Slope factor**	7.5±0.3 (5)	**10.0±0.3 (5)** *
	**V_0.5_ SSI**	−56.3±0.3 mV (5)	−56.6±0.4 mV (5)
	ka **Slope factor**	7.5±0.2 (5)	8.4±0.4 (5)
	**τ Recovery**	0.34±0.01ms (5)	**0.22±0.01 (5)** *
**K_V_1.7**	**V_0.5_ activation**	−10.5±0.6 mV (7)	-
	ka **Slope factor**	9.9±0.5 (7)	-
**K_V_1.7-Ca_V_3.3^DI^**	**V_0.5_ activation**	91.3±0.4 mV (5)	88.2±0.3 mV (5)
	ka **Slope factor**	1.0±0.3 (5)	11.8±0.2 (5)
**K_V_1.7Ca_V_3.3^DII^**	**V_0.5_ activation**	97.3±0.4 mV (9)	**112.6±0.4 mV (7)** *
	ka **Slope factor**	14.1±0.3 (9)	**12.3±0.4 (7)** *
**K_V_1.7-Ca_V_3.3^DIII^**	**V_0.5_ activation**	71.3±0.8 mV (7)	74.7±0.9 mV (5)
	ka **Slope factor**	21.1±0.7 (7)	22.0±0.8 (5)
**K_V_1.7-Ca_V_3.3^DIV^**	V_0.5_ activation	−60.8±0.8 mV (5)	−60.8±0.4 mV (5)
	ka **Slope factor**	10.1±0.7 (5)	10.0±0.4 (5)

* Significant determined from paired t-test with significance threshold set to p<0.05.

**Figure 3.**
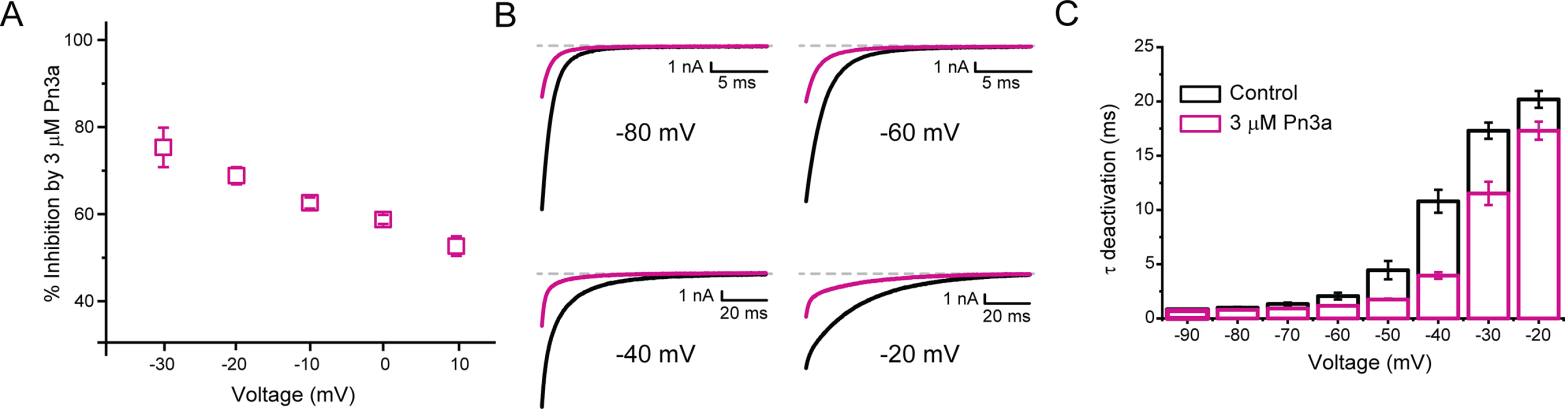
Pn3a inhibition of Ca_V_3.3 is stronger at hyperpolarized potentials and speeds up channel deactivation. (**A**) Voltage dependence of Pn3a inhibition of Ca_V_3.3-mediated Ca^2+^ currents. (**B**) Representative Ca_V_3.3 tail currents at different voltages in the absence (black) and presence (pink) of 3 µM Pn3a. (**C**) Summary of the time constant (τ) of Ca_V_3.3 deactivation upon return to the holding potential (−90 mV) plotted against the activating (pre-pulse) potential in the absence (control, black) and the presence of Pn3a (3 µM, pink). Data shown as mean ± SEM (n=5). Figure 3—source data 1.Voltage dependence of Pn3a inhibition.

The voltage dependence of Pn3a inhibition of I_Ca_ and Na^+^-mediated (I_Na_) Ca_V_3.3 currents was examined at various test potentials (Figure 3A and Figure 3—figure supplement 1). Pn3A displayed a decrease in Ca_V_3.3 I_Ca_ inhibition at more depolarized potentials consistent with Pn3a preferentially binding to the down-state of the VS. To examine the voltage dependence across a larger voltage range, extracellular Ca^2+^ was removed, permitting Na^+^ to be the primary charge carrier through the open Ca_V_3.3. Similar to that seen for I_Ca_, I_Na_ showed a similar voltage dependence, with stronger inhibition at more negative potentials (Figure 3—figure supplement 1).

**Figure 3—figure supplement 1.**
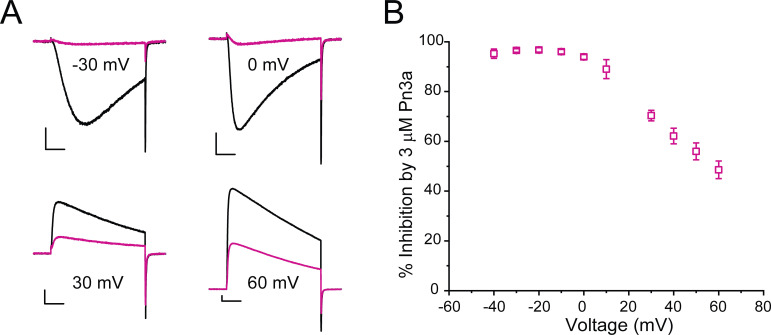
Voltage dependence of Pn3a inhibition of human Ca_V_3.3 when Na^+^ is the charge carrier. (**A**) Representative traces showing the inhibition by 3 µM Pn3a of inward and outward I_Na_ through Ca_V_3.3 at −30, 0, 30, and 60 mV. Scale bars: 1 nA, 20 ms. (**B**) Graph showing % inhibition of I_Na_ by 3 µM Pn3a from −40 to +60 mV.

The time course of tail current decay reflects the rate of channels leaving the open state (test potential) and entering the closed state (deactivation) upon return to the holding potential. We examined the modulation of Ca_V_3.3 channel deactivation through tail current kinetic analysis. Pn3a-modified Ca_V_3.3 currents displayed a faster deactivation time constant (τ_deactivation_) across all potentials tested compared to control (Figure 3B–C). This data suggests that Pn3a binding may destabilize the open state or stabilize the closed one. This results that Pn3a is a Ca_V_3.3 GMP that decreases channel availability by increasing the energy required for channel opening.

### Pn3a interacts with Ca_V_3.3^DII^ S3-S4 paddles

**Figure 4.**
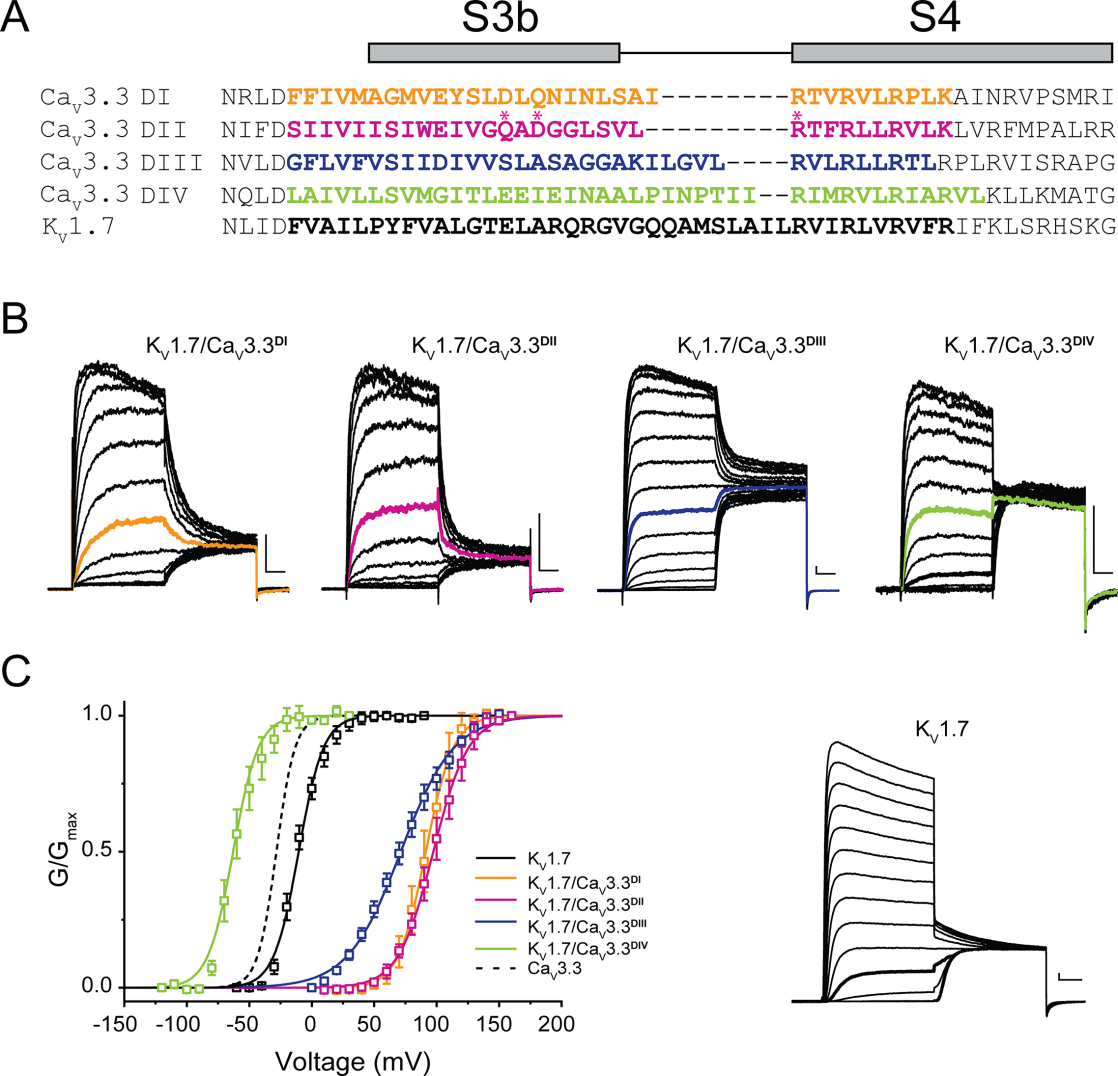
Chimeric constructs of the K_V_1.7 channel with the Ca_V_3.3 voltage sensor paddles. (**A**) Sequence alignment between paddle regions of Ca_V_3.3 and K**_V_**1.7. The coloured bolded sequences from Ca_V_3.3 DI (yellow), Ca_V_3.3 DII (pink), Ca_V_3.3 DIII (blue), and Ca_V_3.3 DIV (green) were grafted onto K**_V_**1.7 (black). (**B**) Representative current traces in response to 50 ms long I-V protocols used to evaluate the voltage dependence of activation of all constructs (Vh = −80 mV). K**_V_**1.7/Ca_V_3.3^DI^ (0–160 mV), K**_V_**1.7/Ca_V_3.3^DII^ (0–160 mV), K**_V_**1.7/Ca_V_3.3^DIII^ (0–160 mV) and K**_V_**1.7/Ca_V_3.3^DIV^ (−120 to 30 mV). The traces highlighted in colour correspond to currents near half-activation potential (V_0.5_). (**C**) Activation curves for all chimeras: K**_V_**1.7/Ca_V_3.3^DI^ (yellow), K**_V_**1.7/Ca_V_3.3^DII^ (pink), K**_V_**1.7/Ca_V_3.3^DIII^ (blue), and K**_V_**1.7/Ca_V_3.3^DIV^ (green) and the parental channel K**_V_**1.7 (black). The dotted line corresponds to Ca_V_3.3 activation for reference. The inset contains representative K**_V_**1.7 currents (−60 to 90 mV). All scale bars: 1 nA, 10 ms.

To ascertain Pn3a’s pharmacophore on Ca_V_3.3 channels, we applied the chimeric approach of transplanting its four S3-S4 paddles into a K_V_ channel based on a similar template to that previously described for K_V_2.1/Ca_V_3.1 chimeras (Salari et al., 2016). We substituted portions of the S3b-S4 from Ca_V_3.3 DI to DIV into the K_V_1.7 channel backbone. The sequence alignment of Ca_V_3.3 DI-DIV S3-S4 segments and the corresponding extracellular region of K_V_1.7 is presented in Figure 4A.

All four K_V_1.7/Ca_V_3.3 chimeric constructs were functional, mediating large K^+^ currents that displayed distinct gating properties from those of the parental wild-type K_V_1.7 (V_0.5_ = −10.5±0.6 mV, n=7; Figure 4B, inset). Briefly, chimeric K_V_1.7/Ca_V_3.3^DI^, K_V_1.7/Ca_V_3.3^DII^ and K_V_1.7/Ca_V_3.3^DIII^ displayed 80–100 mV depolarizing shifts in channel activation (K_V_1.7/Ca_V_3.3^DI^ V_0.5_=91.3 ± 0.4 mV, n=5; K_V_1.7/Ca_V_3.3^DII^ V_0.5_=97.3 ± 0.4 mV, n=9; K_V_1.7/Ca_V_3.3^DIII^ V_0.5_=71.3 ± 0.8 mV, n=7, two-way ANOVA, p<0.05), whereas K_V_1.7/Ca_V_3.3^DIV^ activation was ~50 mV hyperpolarized (K_V_1.7/Ca_V_3.3^DIV^ V_0.5_ = −60.8±0.8 mV, n=5; two-way ANOVA, p<0.05) (Figure 4). Our results are in broad agreement with previous reports for Ca_V_3.1, where DIV displays a hyperpolarizing shift and DI-III show a variable depolarizing shift in V_0.5_ (Salari et al., 2016).

**Figure 5.**
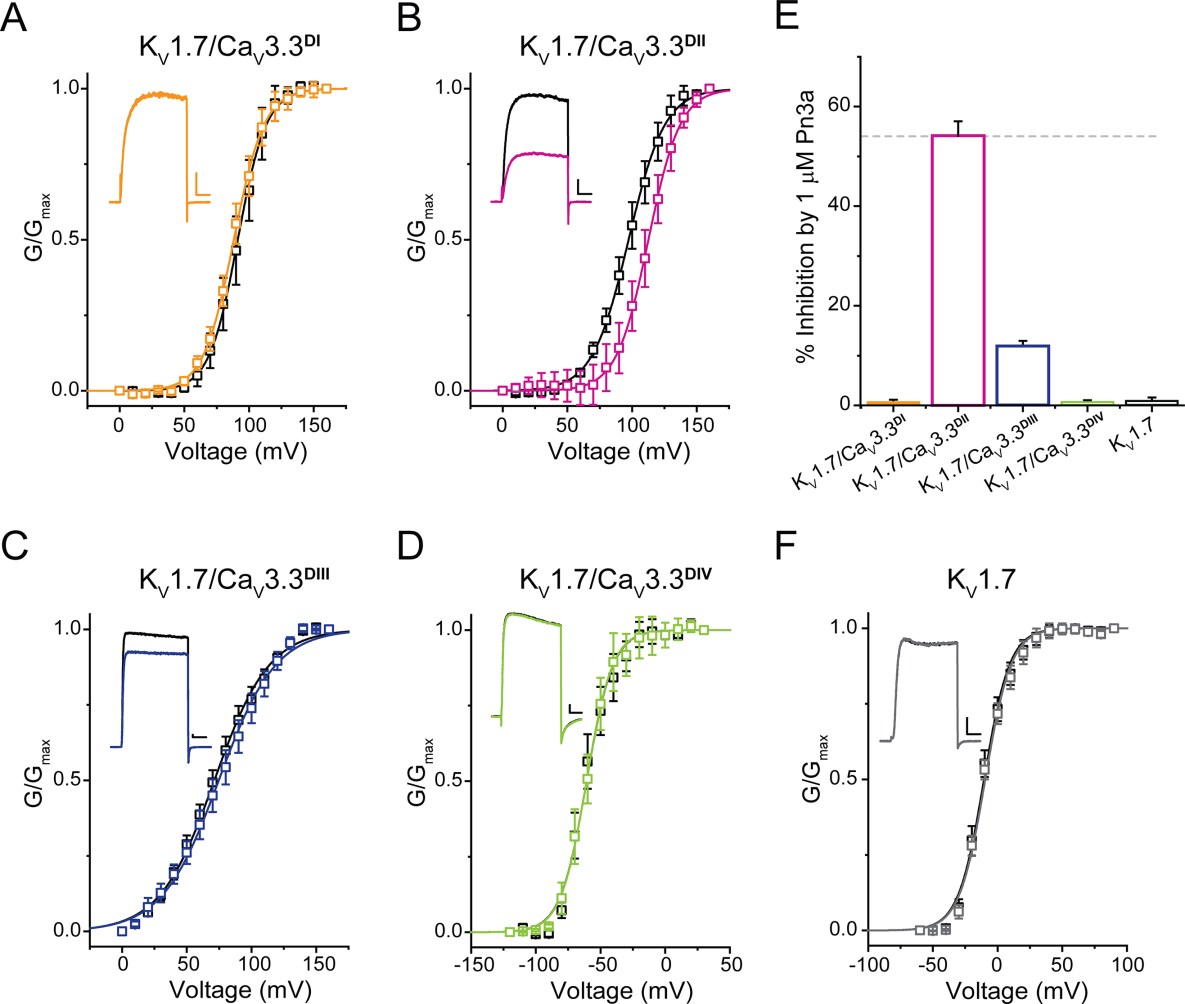
Pn3a shifts the activation of K_V_1.7/Ca_V_3.3^DII^ chimeric channels. G/G_max_-V relationships and representative currents obtained in the absence (control, black) and presence of 1 µM Pn3a (pink) for (**A**) K**_V_**1.7/Ca_V_3.3^DI^ (90 mV), (**B**) K**_V_**1.7/Ca_V_3.3^DII^ (100 mV), (**C**) K**_V_**1.7/Ca_V_3.3^DIII^ (70 mV), and (**D**) K**_V_**1.7/Ca_V_3.3^DIV^ (−60 mV). (**E**) Bar graph showing percent inhibition by 1 µM Pn3a of K**_V_**1.7/Ca_V_3.3^DI-IV^ chimeras and K**_V_**1.7 wt. (**F**) K**_V_**1.7 G/G_max_-V relationship and current traces obtained in the absence (control) and presence of Pn3a (–10 mV). All scale bars: 1 nA, 10 ms. Figure 5—source data 1.Voltage dependence of Pn3a inhibition of Kv1.7/Cav3.3 Chimera.

K_V_1.7/Ca_V_3.3^DI-IV^ chimeras and the parental K_V_1.7 construct were analysed in control and after exposure to Pn3a (1 µM) and representative currents (Vh = −100 mV, with test pulse to +40 mV (K_V_1.7), +120 mV (K_V_1.7/Ca_V_3.3^DI-III^), or 0 mV (K_V_1.7/Ca_V_3.3^DIV^)) in both conditions are included as insets (Figure 5). Similar to the parental channel, peak currents of K_V_1.7/Ca_V_3.3^DI^ and K_V_1.7/Ca_V_3.3^DIV^ chimaeras were insensitive to Pn3a (K_V_1.7: 1.0% ± 0.8%, n=6; Ca_V_3.3^DI^: 0.6% ± 0.5%, n=5, and Ca_V_3.3^DIV^: 0.4% ± 0.2%, n=5) (Figure 5A, D and E), with K_V_1.7/Ca_V_3.3^DIII^ displaying modest peptide-dependent inhibition (12.0% ± 0.6%, n=6) (Figure 5C).

**Figure 5—figure supplement 1.**
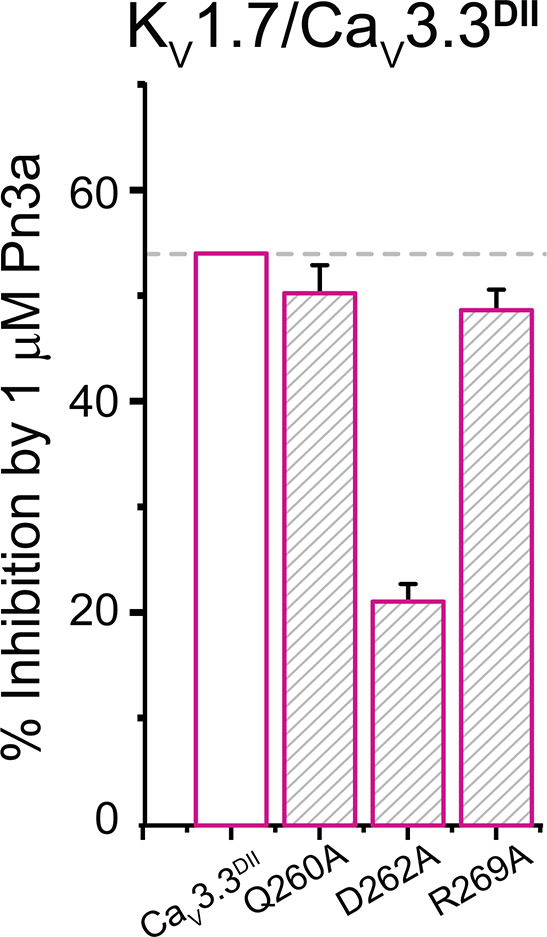
Bar graph showing percent inhibition by 1 µM Pn3a of K_V_1.7/Ca_V_3.3^DII^ chimera and mutants K_V_1.7/Ca_V_3.3^DII^-Q260A, K_V_1.7/Ca_V_3.3^DII^-D262A, and K_V_1.7/Ca_V_3.3^DII^-R269A (corresponding to Ca_V_3.3 −Q687A, −D689A, and −R696A, respectively).

In contrast, currents mediated by K_V_1.7/Ca_V_3.3^DII^ were significantly reduced (54.2% ± 2.9%, n=7) in the presence of Pn3a (Figure 5B). Furthermore, conductance-voltage relationships for each S3-S4 paddle chimera in the absence and presence of Pn3a were built from peak currents in response to standard I-V protocols (K_V_1.7 –60 to +90 mV; K_V_1.7/Ca_V_3.3^DI-III^ 0 to +160 mV; and K_V_1.7/Ca_V_3.3^DIV^ −120 to +30 mV; Vh = −100 mV, 0.2 Hz). The latter analysis revealed an ~15 mV rightward shift in the half voltage of activation (V_0.5_) exclusively in the Ca_V_3.3^DII^ chimera when exposed to Pn3a (control V_0.5_=97.3 ± 0.4 mV, n=9; vs. Pn3a V_0.5_=112.6 ± 0.4 mV, n=7, p<0.0001), strongly suggesting that Pn3a interacts with the S3-S4 region of domain II in Ca_V_3.3. These results suggest that the positive shift in activation V_0.5_ observed in the full length and DII chimera likely underpins Pn3a’s mechanism of Ca_V_3.3 inhibition, highlighting the differences in pharmacological properties of each voltage-sensing module.

### Molecular determinants of Pn3a inhibition of Ca_V_3.3

**Figure 6.**
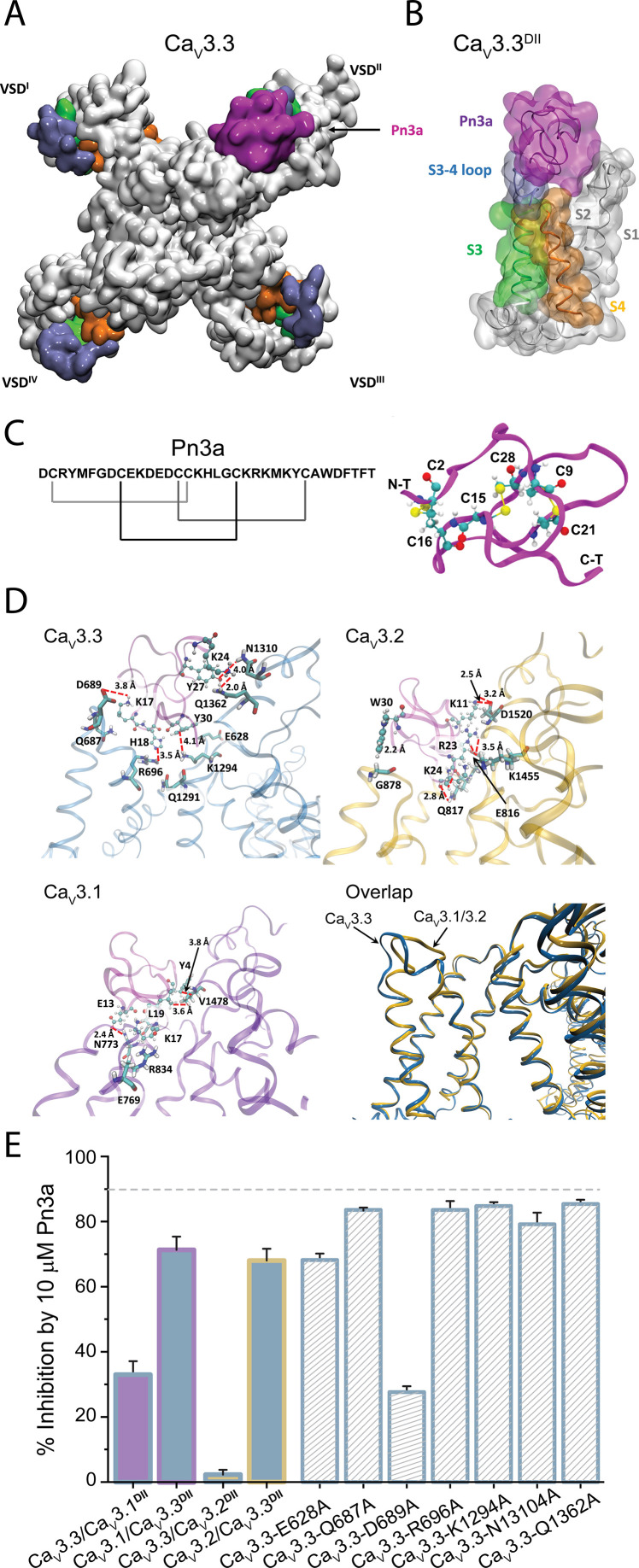
Pn3a and Ca_V_3.3 interactions and subtype specificity. (**A**) Top view of Pn3a (magenta) binding to Ca_V_3.3^DII^. The S3 (lime), S3-4 loop (ice blue), and S4 (pink) are highlighted. (**B**) Side view of the binding conformation of Pn3a to Ca_V_3.3^DII^. (**C**) Left: Pn3a amino acid sequence and disulfide connectivity. Right: 3D structure of Pn3a (PDB ID: 5T4R Deuis et al., 2017) showing cysteine residues in CPK linked by salt bridges (yellow). (**D**) Pairwise amino acid interactions between Pn3a (magenta) with the extracellular loop of Ca_V_3.3^DII^ (top left, blue), Ca_V_3.2^DII^ (top right, yellow), and Ca_V_3.1^DII^ (bottom left, purple). Overlap of the Pn3a-bound ribbon structures of all Ca_V_3 highlighting differences in extracellular S3-S4 loops between Ca_V_3.3 and the other two Ca_V_3 isoforms (bottom right). S1, S1-2 loop, and S2 were removed for clarity. (**E**) Bar graph showing percent inhibition by 10 µM Pn3a of Ca_V_3^DII^ domain-swapped Ca_V_3 constructs and point mutants. Figure 6—source data 1.Pn3a docking coordinates for T-type calcium channels.

The critical role of DII was further supported by examining the effects of Pn3a on full-length Ca_V_3 channel constructs where the whole DII was swapped between isoforms. Replacement of Ca_V_3.3^DII^ with either Ca_V_3.1^DII^ or Ca_V_3.2^DII^ resulted in partial (Ca_V_3.3/Ca_V_3.1^DII^: 33% ± 4.1%, n=5, one-way ANOVA p<0.0001) or complete (Ca_V_3.3/Ca_V_3.2^DII^: 2.2% ± 1.5%, n=5, one-way ANOVA p<0.0001) loss of inhibition compared to the full-length Ca_V_3.3 (90.2 ± 1.9% n=5) in the presence of 10 μM Pn3a (Figure 6E). The reverse constructs where Ca_V_3.3^DII^ was grafted onto Ca_V_3.1 (Ca_V_3.1/Ca_V_3.3^DII^) or Ca_V_3.2 (Ca_V_3.2/Ca_V_3.3^DII^) afforded Pn3a sensitivity to the parental Ca_V_3 channel such that currents mediated by Ca_V_3.1/Ca_V_3.3^DII^ were inhibited by 71.4% ± 4.1% (n=5), and Ca_V_3.2/Ca_V_3.3^DII^ by 68.0% ± 3.6% (n=5) (Figure 6F). Thus, verifying that Ca_V_3.3^DII^ is required for Pn3a interaction with T-type calcium channels.

**Figure 6—figure supplement 1.**
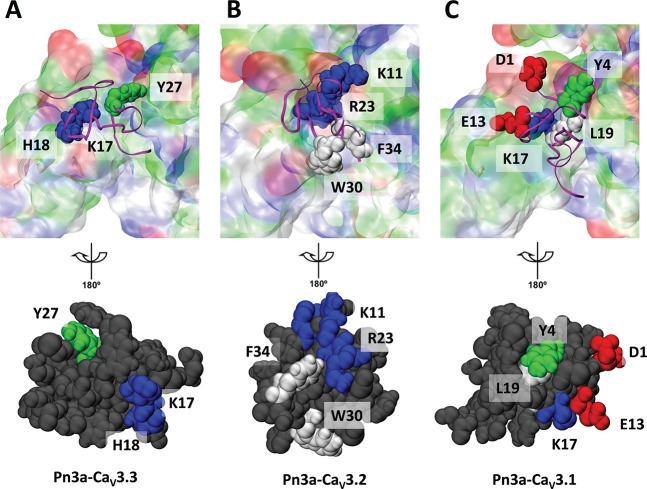
The binding conformation of Pn3a at voltage-sensing domain (VSD)-II of T-type calcium channels. The interactive residues of the spider toxin contacting with corresponding residues of the VSDII of Ca_V_3.3 (**A**), Ca_V_3.2 (**B**), and Ca_V_3.1 (**C**), respectively. For clarity, the binding domain of Ca_V_3 channel shown in transparent colour was removed after rotating the binding conformation around the y-axis by 180° (in the bottom). The colouring method in visual molecular dynamics (VMD) was adopted for the residues and surfaces, demonstrating non-polar (white), basic (blue), acidic (red), and polar (green) residues.

**Figure 6—figure supplement 2.**
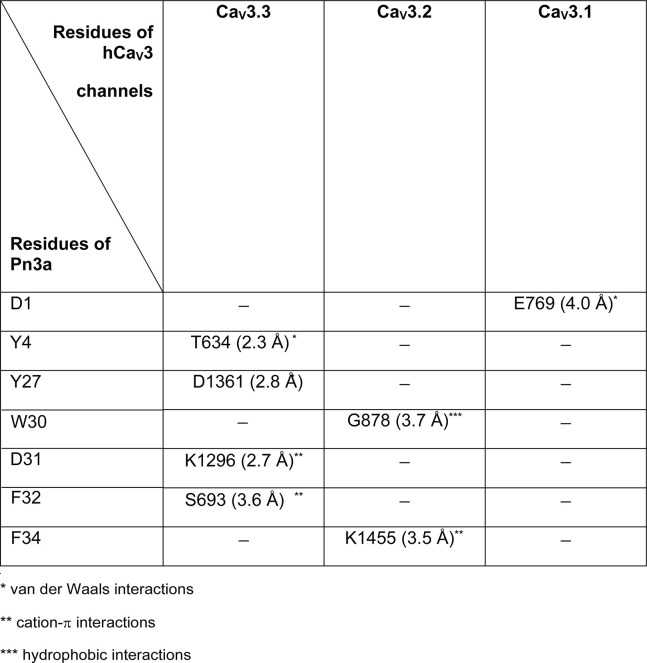
Minor interactions between the residues of Pn3a and the corresponding residues on hCa_V_3 channels.

**Figure 6—figure supplement 3.**
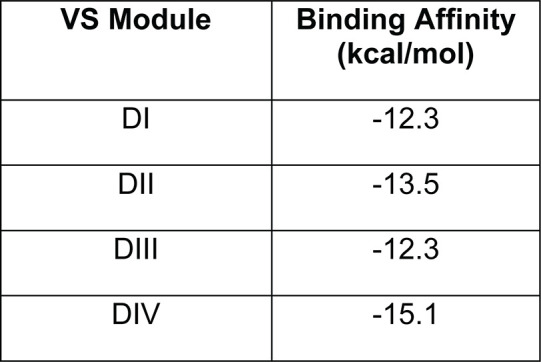
Predicted binding affinity values for Pn3a binding to the DI, DII, DIII, and DIV domains of the voltage sensor (VS) modules on hCa_V_3.3 calculated using Autodock Vina.

Molecular docking was used to assess the most energetically favoured binding poses for Pn3a on the extracellular-facing pockets of DII of the three channel isoforms, Ca_V_3.3, Ca_V_3.2, and Ca_V_3.1 (Figure 6 and Figure 6—figure supplements 1–3). Comparative analyses of toxin interactions with the other T-type calcium channel members showed that Pn3a forms fewer contacts with the DII S3-S4 linker of Ca_V_3.1 and Ca_V_3.2, compared to Ca_V_3.3 (Figure 6D). Evaluation of the inter-residue contacts between the docked Pn3a and Ca_V_3.3^DII^ revealed several receptor residues predicted to form close contacts with the toxin (Figure 6D, upper left inset). In particular, Pn3a-K17 is predicted to be in close proximity to D689 and may form an especially strong interaction due to the possibility of a salt bridge contact. Guided by the docking results, point mutations of selected Cav3.3^DII^ amino acids (E628A, Q687A, D689A, R696A, K1294A, N1310A, and Q1362A) were explored within full-length Cav3.3 channels. In comparison to wild-type Ca_V_3.3, Ca_V_3.3-D689A (27.6% ± 1.8%, n=5, one-way ANOVA p<0.0001) and Ca_V_3.3-E628A (68.2% ± 2.0%, n=5, one-way ANOVA p<0.0001) displayed significant reductions in Pn3a inhibition (Figure 6F), whereas the peptide activity in the other mutants studied (Ca_V_3.3-Q687A: 83.6% ± 0.5%, n=5; Ca_V_3.3-R696A: 83.6% ± 2.7%, n=5; Ca_V_3.3-K1294A: 84.8% ± 1.2%, n=5; Ca_V_3.3-N1310A 79.2% ± 3.5%, n=5; and Ca_V_3.3-Q1362A 85.4% ± 1.3%, n=5) was unaffected. The relevance of Ca_V_3.3-D689 interaction with Pn3a was further substantiated by the loss of inhibition in the analogous charge neutralization K_V_1.7/Cav3.3^DII^ chimeric construct K_V_1.7/Ca_V_3.3^DII^-D262A (Figure 5—figure supplement 1), thus pinpointing key molecular determinants responsible for Pn3a’s selective inhibition of Ca_V_3.3.

## Discussion

The present study demonstrates the functional activity of Pn3a as a gating modifier inhibitor of human Ca_V_3.3 channels with >100-fold higher activity over Ca_V_3.1 and Ca_V_3.2. Our chimeric approaches revealed Pn3a’s preference for Ca_V_3.3^DII^ S3-S4 paddle region, whereas comparative molecular docking amongst isoforms identified a novel binding site putatively determining Pn3a’s proclivity towards Ca_V_3.3. Thus, this investigation highlights Pn3a as the first molecular probe available for the study of Ca_V_3.3 contribution in native cells. We propose that the unique features of Pn3a’s Ca_V_3.3 specificity may be exploited to design isoform-selective T-type calcium channel modulators.

### Ca_V_3 subtype selectivity

µ-Theraphotoxin Pn3a was originally described as a potent Na_V_1.7 inhibitor with an IC_50_ of 0.9 nM, while also inhibiting other tetrodotoxin-sensitive Na_V_ channels (Na_V_1.1–1.4 and Na_V_1.6) with 10- to 100-fold less potency (Deuis et al., 2017). More recently Pn3a was shown to inhibit murine dorsal root ganglion HVA I_Ca_ and human Ca_V_1.2, Ca_V_1.3, Ca_V_2.1, and Ca_V_2.2 calcium channels expressed in HEK293T cells (IC_50_ 3–10 μM) (McArthur et al., 2020). Perhaps a more interesting aspect of Pn3a activity is its >100-fold selectivity for human Ca_V_3.3 over both Ca_V_3.1 and Ca_V_3.2 (Figure 1) which distinguishes this spider peptide as a unique isoform-selective modulator of T-type calcium channels. Only a handful of venom-derived peptides have been shown to interact with T-type calcium channels. The scorpion peptide Kurtoxin was the first GMP reported to modulate Ca_V_3 channels with a high affinity for Ca_V_3.1 and Ca_V_3.2 channels (Chuang et al., 1998). The closely related peptide KLI, from *Parabuthus granulatus*, was later shown to inhibit Ca_V_3.3 with an IC_50_ ~ 450 nM (Olamendi-Portugal et al., 2002). However, the T-type isoform selectivity and binding site of these two peptides were not comprehensively documented at the time. The activity of spider Protoxins I and II against the three Ca_V_3 channels revealed that ProTx-I preferentially modulates Ca_V_3.1 channels, whereas ProTx-II targets Ca_V_3.2 (Bladen et al., 2014; Salari et al., 2016). Thus, Pn3a complements the molecular toolbox for the study of T-type calcium channels in native tissues.

### Voltage dependence and current kinetics

Pn3a inhibits Ca_V_3.3 by inducing a depolarizing shift in Ca_V_3.3 voltage dependence of activation in a manner analogous to Kurtoxin, Protoxin I, and Protoxin II actions on Ca_V_3.1 channels (Edgerton et al., 2010; Chuang et al., 1998). This is also consistent with Pn3a’s modulation of Na_V_1.7 (Deuis et al., 2017) but not of Ca_V_2.2 channels for which a hyperpolarizing shift in the voltage dependence of inactivation was associated with current inhibition (McArthur et al., 2020). In contrast to the twofold slower recovery from inactivation reported for Pn3a-bound Na_V_1.7 channels, currents mediated by Pn3a-modified Ca_V_3.3 channels recover ~1.5-fold faster from inactivation (Figure 2D/E). This and the observed slowing of inactivation (at high Po) are suggestive of a toxin-induced destabilization of the inactivated state. Nevertheless, given Pn3a’s voltage dependence of Cav3.3 inhibition (Figure 3A), voltage-dependent unbinding of the toxin at more positive potentials could also contribute to an apparent faster recovery from inactivation as reported for Ca_V_ active toxins such as AgaIVA (McDonough et al., 1997b) and SNX482 (Bourinet et al., 2001).

Pn3a inhibition of Ca_V_3.3 was voltage-dependent with greater inhibition at more negative potentials (Figure 3A) similar to Kurtoxin-inhibited Ca_V_3.1 channel currents (Chuang et al., 1998). However, a delay in Ca_V_3.1 activation kinetics was apparent in the presence of both Kurtoxin and ProTx-II (Edgerton et al., 2010; Chuang et al., 1998) but not in Pn3a-modified Ca_V_3.3. The spider peptides Pn3a (this study) and ProTx-II (Edgerton et al., 2010) appear to slow T-type channel deactivation suggestive of stabilization of the channel’s closed state, whereas Kurtoxin, from scorpion venom, does not affect Ca_V_3.1 channel closure (Chuang et al., 1998) highlighting incompletely understood aspects of GMP/VGIC interactions.

### Interaction of Pn3a with Ca_V_3.3 VS paddles

The portability of the S3-S4 paddle region was shown more than 20 years ago (Swartz and MacKinnon, 1997). To date, most studies, if not all, have used the K_V_2.1 channel backbone for the identification of GMPs binding sites in Na_V_ and Ca_V_ channels expressed in *Xenopus* oocytes (Bosmans et al., 2008; Salari et al., 2016). For our chimera studies, we selected the K_V_1.7 backbone given the remarkable scarcity of GMPs interacting with K_V_1 channels (Finol-Urdaneta et al., 2020). The robust expression of this channel in heterologous systems (Finol-Urdaneta et al., 2006; Finol-Urdaneta et al., 2012) and resistance to Pn3a modulation (Figure 5E/F) make it suitable to assess peptide binding to Ca_V_3.3 paddle motifs in mammalian cells. The generated K_V_1.7/Ca_V_3.3 constructs resulted in four functional, voltage-gated chimeric potassium channels that exhibited distinct gating properties to those of the parental scaffold reflecting the acquisition of the grafted S3-S4 paddle regions from DI to DIV of Ca_V_3.3. Namely, K_V_1.7/Ca_V_3.3^DI-DIII^ all displayed >80 mV depolarizing shifts in channel activation compared to wild-type K_V_1.7, whereas the Ca_V_3.3^DIV^ bearing chimeric construct presented a comparable magnitude shift in the hyperpolarizing direction (Figure 4).

Analogous chimeric approaches of K_V_2.1/Ca_V_3.1D^I-IV^ have indicated that ProTx-II, PaTx-1, GsAF-I, and GsAF-II exert their inhibitory actions predominantly through interaction with Ca_V_3.1^DIII^ (Salari et al., 2016), whereas Pn3a does so by targeting K_V_2.1/Na_V_1.7^DII^ and K_V_2.1/Na_V_1.7^DIV^ (Deuis et al., 2017). Here, we observe modest Pn3a inhibition of K_V_1.7/Ca_V_3.3^DIII^-mediated currents and potent effects on K_V_1.7/Ca_V_3.3^DII^ chimeras with current inhibition coupled to toxin-induced rightward shift in the voltage dependence of activation as evidence of preferential interactions with Ca_V_3.3^DII^ (Figure 5B/F). A substantial body of literature has shown that DI-DIII and DIV are important for Na_V_ activation and inactivation, respectively (Ahern et al., 2016). Thus, the predominant effects of Pn3a on Ca_V_3.3 activation are consistent with its interaction with DII of this channel.

Furthermore, it has been shown that ProTx-I inhibits Ca_V_3.3/Ca_V_3.1^DIV^ chimeric channels while interacting less potently with Ca_V_3.3/Ca_V_3.1^DII^. However, a clear binding site could not be delineated through mutation of individual Ca_V_3.1^DII^ residues as those did not result in measurable changes in toxin affinity (Bladen et al., 2014). Our domain swap experiments verified that Pn3a’s actions are largely determined by Ca_V_3.3^DII^ from which aspartate in position 689 constitutes an important interaction site as shown by its alanine replacement in Ca_V_3.3 and K_V_1.7/Ca_V_3.3^DII^. It can be surmised that subtle, but significant GMP/VGIC interaction differences highlight incompletely understood idiosyncrasies related to molecular aspects of ion channel function between isoforms as well as how peptide interactions may be affected by the experimental manipulation and conditions used for their study.

### A novel binding site with Ca_V_3 subtype selectivity

Our molecular docking calculations suggest that Pn3a binds within the groove formed between extracellular linkers S1-S2 and S3-S4 supported by electrostatic interactions with the Ca_V_3.3^DII^ S3-S4 paddle, which bears some similarity with the Ca_V_3.1/ProTx-II complex in which favourable binding sites occur in Ca_V_3.1’s DII and DIV (Bladen et al., 2014).

The interaction of Pn3a with Ca_V_3.3 channels involves substantial interactions within the S3-S4 paddle (K17-Q687) and S4 (H18-R696) of Ca_V_3.3^DII^, while a close contact may also exist between the sidechain of D689 and the sidechain of Pn3a-K17 (Figure 6D, upper left panel). The latter is consistent with the establishment of a salt bridge between these residues. The loss of Pn3a inhibition upon charge neutralization at this position lends support to this docking model.

The proximity between cationic residues (K17 and H18) on loop 3 of Pn3a and the Ca_V_3.3D^II^ S3-S4 paddle places this peptide at the hollow between S1-S2 and S3-S4 linkers in a binding conformation similar to other tarantula toxins targeting Na_V_ channel site 4, like HwTX-IV, ProTx-II, and HNTX-III, that bear critical basic and hydrophobic amino acids within loop 4 that interact with acidic residues on the Na_V_^DII^’s S3-S4 linker (Deng et al., 2013; Liu et al., 2013; Bosmans and Swartz, 2010; Figure 6—figure supplements 1 and 2). Hence, the predicted Pn3a interaction with Ca_V_3.3 appears to share overall similarities with previously identified spider peptide toxins modulating Na_V_ and K_V_ channels in which the common bioactive surface consists of positively charged and hydrophobic residues (Smith et al., 2007; Corzo et al., 2005).

The findings presented here establish Pn3a as a gating modifier modulator of Ca_V_3.3 channels interacting with the paddle motif of DII’s VS module through putative stabilization of the closed/resting state and concomitant channel current inhibition. Pn3a’s>100-fold higher potency against Ca_V_3.3 over the other two Ca_V_3 isoforms is rationalized through recognition of a previously unknown drug binding site that may be exploited in the design of isoform-selective Ca_V_3 channel modulators.

## Materials and methods

### Cell lines, culture, and transfections

Human embryonic kidney (HEK293T, authenticated by STR profiling and mycoplasm free) cells containing the SV40 Large T-antigen were cultured and transfected by calcium phosphate method as reported previously (McArthur et al., 2018). In brief, cells were cultured at 37°C, 5% CO_2_ in Dulbecco’s modified Eagle’s medium (DMEM, Invitrogen Life Technologies, VIC, Australia), supplemented with 10% fetal bovine serum (FBS, Bovigen, VIC, Australia), 1% GlutaMAX and penicillin-streptomycin (Invitrogen). HEK293T cells were then transiently co-transfected with the different Ca_V_ channel isoforms and green fluorescent protein (GFP) for visualization, using the calcium phosphate method. cDNAs encoding human Ca_V_3.1 (provided by Dr G Zamponi), human Ca_V_3.2 (a1Ha-pcDNA3 was a gift from Dr E Perez-Reyes, Addgene #45809) (Cribbs et al., 1998), human Ca_V_3.3 (a1Ic-HE3-pcDNA3 also from Dr E Perez-Reyes, Addgene #45810) (Gomora et al., 2002) in combination with GFP. Ca_V_3^DII^ swap constructs and K_V_1.7/Ca_V_3.3 chimeras were custom synthesized by GeneScript, NJ.

Chinese hamster ovary (CHO) cells were used to express K_V_1.7 and K_V_1.7-Ca_V_3.3 paddle chimeras. Cell culture conditions were the same as the HEK293T cells except DMEM was substituted with DMEM/F12 (Invitrogen). CHO cells were transfected with cDNAs encoding K_V_1.7 and K_V_1.7/Ca_V_3.3^DI-DIV^ chimeras using Lipofectamine 2000 (Invitrogen) as per the manufacturer’s protocol and used for recordings 12–48 hr post-transfection.

### Electrophysiology

Whole-cell patch clamp configuration was used to record calcium (I_Ca_) or potassium (I_K_) currents in transiently transfected HEK293T cells. Recordings were made using a MultiClamp 700B amplifier, digitized with a DigiData1440, and controlled using Clampex11.1 software (Molecular Devices, San Jose, CA). Whole-cell currents were sampled at 100 kHz and then filtered to 10 kHz, with leak and capacitive currents subtracted using a −P/4 protocol for Ca^2+^ currents and uncorrected for K^+^ currents. All recordings were series compensated 60–80%. External solution for I_Ca_ contained in mM: 100 NaCl, 10 CaCl_2_, 1 MgCl_2_, 5 CsCl, 30 TEA-Cl, 10 D-glucose and 10 HEPES, pH 7.3 with TEA-OH. External solution for I_K_ contained in mM: 140 NaCl, 5 KCl, 1 MgCl_2_, 2 CaCl_2_, 10 glucose, and 10 HEPES, pH 7.3 with NaOH. Fire-polished borosilicate (1B150F-4, World Precision Instruments, Sarasota, FL) patch pipettes were used with resistance of 1–3 MΩ. Intracellular recording solution contained as follows (mM): 140 KGluconate, 5 NaCl, 2 MgCl_2_, 5 EGTA, and 10 HEPES, pH 7.2 with KOH. Cells were continuously perfused with extracellular solution at a rate of 1.2 ml/min, while toxin application was superfused onto the cell through a capillary tube attached to a syringe pump (2 µl/min), directly onto the cell being recorded.

For experiments on Ca_V_3s, all cells were held at −90 mV. To examine the onset of block, test pulses (100 ms, 0.5 Hz) to −20 mV were applied. To generate activation curves, cells were pulsed from −90 to +40 mV in 10 mV increments at 0.5 Hz. SSI curves were generated by measuring the peak current from a test pulse to −20 mV when preceded by a 1 s pre-pulse from −90 to 20 mV (0.1 Hz). Recovery from inactivation curves was produced by varying the time (0–2 s) between two depolarizing pules to −20 mV (P1 200 ms, P2 50 ms).

For experiments on K_V_1.7 and Ca_V_3.3 DI-IV chimeras, cells were held at −80 mV and activation curves were generated by applying a 50 ms pre-pulse to varying potentials depending on the channel construct examined (K_V_1.7 –60 to 90 mV, DI-III 0 to +160 mV, and DIV −120 to +30 mV) followed by a 50 ms test pulse to measure tail currents (K_V_1.7 0 mV, DI-III +80 mV, and DIV −20 mV). To measure the onset of Pn3a inhibition, test pulses (50 ms) from a holding potential of −100 mV to a test potential determined for each channel construct (K_V_1.7 40 mV, DI-III 120 mV, and DIV 0 mV) were elicited at 0.2 Hz.

### Structures and homology modelling of human T-type calcium channels and the docking of Pn3a

The cryo-EM structures of human Ca_V_3.1 (PDB ID: 6KZO) (Zhao et al., 2019) and Ca_V_3.3 (PDB ID: 7WLI) (He et al., 2022) were used for docking calculations. The comparative model of humanCa_V_3.2 was built upon the cryo-EM structure of hCa_V_3.1 in the apo form (PDB ID: 6KZO) via the SWISS-MODEL server (https://swissmodel.expasy.org/). The 3D structure of μ-theraphotoxin (TRTX)-Pn3a (PDB ID: 5T4R) (Deuis et al., 2017) was docked into the *three* T-type calcium channels via Autodock Vina with a grid box of 40Å × 40Å × 40 Å. We also compared the binding mode and the binding affinity amongst the four VSs of Ca_V_3.3, to identify which VS module Pn3a preferentially targets. The docking results were further analysed via Discovery Studio 2017R2 and visualized using Visual Molecular Dynamics (VMD) version 1.9.3 (Humphrey et al., 1996).

### Data and statistical analysis

All data analysis and graphs were generated in OriginPro (Origin Lab Corporation, Northampton, MA). Concentration-response curves were generated by plotting peak current amplitudes in the presence of Pn3a (I_Pn3a_), over the current before Pn3a application (I_Control_). The resulting curve was fit with a sigmoidal curve according to the following expression:

(1) $$I_{Pn3a}/I_{Control}=1+{\left[Pn3a\right]}^{n}/\left(IC50^{n}+{\left[Pn3a\right]}^{n}\right)$$

where IC50 is the half-maximal inhibitory concentration and n is the Hill coefficient. Activation (Equation 2) and SSI (Equation 3) curves were fit by the modified Boltzmann equation:

(2) $$I=1-1/\left(1+\exp\left(\frac{Vm-V_{0.5}}{ka}\right)\right)$$

(3) $$G=1/\left(1+\exp\left(\frac{Vm-V_{0.5}}{ka}\right)\right)$$

where I is the current or G is the conductance, V_m_ is the pre-pulse potential, V_0.5_ is the half-maximal activation potential, and ka is the slope factor. Recovery from inactivation plots was fit using a single exponential of the following equation:

(4) $$\left(\frac{P2}{P1}\right)=1+A_{fast}*\exp\left(-\frac{t}{t}\right)$$

where τ is the time constant and A is the amplitude. Statistical significance (p<0.05) was determined using paired or unpaired t-test or two-way ANOVA followed by a Tukey multiple comparison test if F achieves the level of statistical significance of p<0.05 and no variance inhomogeneity. All data are presented as mean ± SEM (n), where n is individual cells with all experimental results containing n≥5 individual cells.

## Data Availability

All data generated or analysed during this study are included in the manuscript and supporting file.

## Additional files

Transparent reporting form
